# Endobronchial Valves for the Treatment of Advanced Emphysema

**DOI:** 10.1016/j.chest.2020.12.007

**Published:** 2020-12-17

**Authors:** Karin Klooster, Dirk-Jan Slebos

**Affiliations:** University of Groningen, University Medical Center Groningen, Department of Pulmonary Diseases, The Netherlands

**Keywords:** bronchoscopy, COPD, emphysema, endobronchial valve, lung volume reduction, BLVR, bronchoscopic lung volume reduction, Dlco, diffusing capacity for carbon monoxide, EBV, endobronchial valve, FCS, fissure completeness score, HRCT, high resolution CT, LVR, lung volume reduction, QCT, quantitative CT, RV, residual volume

## Abstract

Bronchoscopic lung volume reduction with one-way endobronchial valves is a guideline treatment option for patients with advanced emphysema that is supported by extensive scientific data. Patients limited by severe hyperinflation, with a suitable emphysema treatment target lobe and with absence of collateral ventilation, are the responders to this treatment. Detailed patient selection, a professional treatment performance, and dedicated follow up of the valve treatment, including management of complications, are key ingredients to success. This treatment does not stand alone; it especially requires extensive knowledge of COPD for which the most appropriate treatment is discussed in a multidisciplinary approach. We discuss the endobronchial valve treatment for emphysema and provide a guideline for patient selection, treatment guidance, and practice tools, based on our own experience and literature.

Bronchoscopic lung volume reduction (BLVR) with one-way endobronchial valve (EBV) is a minimally invasive treatment that has been shown to improve clinical outcomes in patients with advanced emphysema and severe hyperinflation.^1^^,^^2^ Bronchoscopic placement of EBVs in a suitable target lobe, with proven absence of collateral ventilation with the use of the Chartis Pulmonary Assessment System (Pulmonx Corporation, Redwood City, CA),^3^ induces a (partial) atelectasis of the target lobe.^4^ This atelectasis ensures reduction in residual volume (RV) and results in an improvement in lung function, a greater exercise performance, and a better quality of life (Table 1).^1^^,^^5^, ^6^, ^7^

**Table 1:** Results of the Randomized Controlled Trials Investigating Zephyr Endobronchial Valve^a^ Treatment in Patients With Severe Emphysema With Proven Absence of Collateral Ventilation With the Use Chartis Assessment System^a^

Variable	Trial			
	STELVIO^1^	IMPACT^7^	TRANSFORM^6^	LIBERATE^5^
Patients, No./No.	EBV34/SoC34	EBV43/SoC50	EBV65/SoC32	EBV128/SoC62
Follow up, mo	6	3	6	12
Target lobar volume reduction, mL	−1366	−1195	−1090	−1142
Between group difference				
FEV_1_, %	+18	+17	+29	+18
Residual volume, mL	−831	−480	−700	−522
6-Minute walk distance, m	+74	+40	+79	+39
St. George’s Respiratory Questionnaire, points	−14.7	−9.7	−6.5	−7.1

EBV = Zephyr endobronchial valve; SoC = Standard of care.

a Pulmonx Corporation, Redwood City, CA.

Almost two decades ago, the very first EBV case series were published.^8^^,^^9^ Based on a large number of subsequent randomized controlled trials,^1^^,^^5^, ^6^, ^7^^,^^10^^,^^11^ EBV treatment has regulatory approval (CE-Mark; in the United States, Food and Drug Administration^12^) and is now a GOLD-COPD^13^ and United Kingdom-NICE^14^ guideline treatment in patients with advanced emphysema.^15^ Because EBV treatment is a treatment for a very difficult to treat highly prevalent disease, there is an increasing worldwide demand to offer this treatment to patients with severe COPD. In this “How I do it” review, we provide a deeper insight by sharing our in-house expertise to new and also more experienced programs to ensure continued success with this exciting treatment.

## Case Example

**Figure 1:**
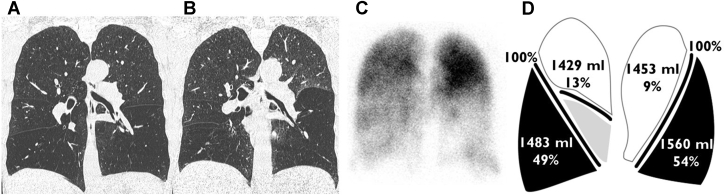
A-D, Case example of a suitable patient with COPD for bronchoscopic lung volume reduction with the use of one-way endobronchial valves. **A**, Inspiration chest CT scan shows lower lobe predominant heterogeneous emphysema; B, expiration chest CT scan shows predominant air trapping in the left lower lobe; C, perfusion scan confirms the left lower lobe target by absence of perfusion; D, quantitative CT analysis shows percentage of emphysematous destruction (at −950 Hounsfield Units), lobar volumes, and fissure integrity.

A 58-year-old woman with COPD (FEV_1_, 30% of predicted; RV, 239% of predicted), ex-smoker, is receiving optimal medication and recently performed a pulmonary rehabilitation program. Despite all this, the patient still experiences severe dyspnea, a limited exercise capacity, and a poor quality of life. Chest imaging showed that the left lower lobe is a good treatment target lobe for BLVR with the use of valves (Fig 1). Chartis assessment confirmed the absence of collateral ventilation in the target lobe, and a total of 5 EBVs were placed into the left lower lobe. Total procedure time was 30 minutes; no complications occurred. The patient was discharged three nights after the procedure. At 1 year after treatment, she continued to perceive less dyspnea, increased her 6-minute walk distance by 30%, and experienced a better quality of life without side-effects. The high-resolution CT (HRCT) scan showed a complete atelectasis of the left lower lobe with a decrease in RV of 1044 mL and FEV_1_ improvement of 41% compared from baseline.

## How Do I Select Patients?

Selecting patients for lung volume reduction (LVR) therapies is all about COPD phenotyping^16^ and is kind of the “art of balancing.” Balancing between reducing hyperinflation and maintaining sufficient gas-exchange, balancing between the volume of the lobe to be treated vs the ipsilateral nontreated lobe, and balancing between the expected potential benefit and the risks involved. Thus the question is “how do I know if my patient is a suitable candidate for EBV treatment?” Potential patients who have advanced COPD remain highly symptomatic despite receiving optimal medical treatment.^13^^,^^14^ To facilitate this question a structured step-wise evaluation approach can be very helpful.

### Step 1: Symptoms and Limitations

Symptoms can be assessed with validated questionnaires. Symptomatic patients with COPD Assessment Test^17^ scores ≥10 and modified Medical Research Council dyspnea scale dyspnea score^18^ ≥2, and limited exercise performance (6-minute walk distance between 100 and 450 m) are candidates.

LVR candidates should be limited significantly because of lung hyperinflation. Assessment of symptoms and physical limitations is, however, very subjective and also largely dependent on individual patient preferences.^19^ It is important to understand and discuss a patient’s treatment outcome expectations and goals and to explain the pros and cons of not performing a treatment or alternative options. Patient preference is an important topic of any LVR program, because the treatments performed are often timed in an end-of-life situation of the patient with COPD and often will result in the desired outcome but can also end in disappointment.^19^^,^^20^

### Step 2: Optimal Medical Treatment

Smoking cessation, receiving guideline pharmacologic therapy, completed pulmonary rehabilitation, and/or are participating in a structured physical therapy program, nutrition support, long-term oxygen therapy, and noninvasive ventilation should all have been evaluated and optimized as appropriate.^13^^,^^21^

### Step 3: Airflow Obstruction

Postbronchodilator FEV_1_/FVC < 70% and FEV_1_ between 15% and 50% of predicted are suggestions. Good outcomes have been published in selected cases with very optimal treatment targets that had both lower and higher FEV_1_ values.^1^^,^^22^ The bottom line is that patients need to have severe COPD that is proven to be the limiting factor of their dyspnea.

### Step 4: Hyperinflation

Postbronchodilator RV ≥175% of predicted and RV/total lung capacity ≥55%, measured by body plethysmography are suggestions. Good outcomes have been published in selected cases with very optimal treatment targets that had a lower RV or RV/TLC ratio.^23^ As long as an individual patient is significantly limited by hyperinflation, the patient can be evaluated for LVR options.

### Step 5: Comorbidities

Patients are less likely to be eligible if they have one of more of the following occurrences:

• Severe hypercapnia (Paco_2_ >8 kPa/>60 mm Hg) or severe hypoxia (Pao_2_ <6.0 kPa/>45 mm Hg) at room air (sea level), both for safety reasons
• Significant congestive heart failure (left ventricular ejection fraction <40%)
• Pulmonary hypertension (right ventricular systolic pressure >50 mm Hg)
• Use of coumadins/antiplatelets, which cannot be stopped around the procedure
• Maintenance immunosuppressive agents or prednisolone ≥10 mg daily (to avoid severe local microbiologic colonization of the device)
• Previous lobectomy, lung transplantation, or LVR surgery
• Frequent infectious exacerbations (bronchitis phenotype) and/or symptomatic bronchiectasis
• Diffusing capacity for carbon monoxide (Dlco) <20% or >60% of predicted

A Dlco >60% means that there is still well lung tissue preserved, which is another COPD phenotype. Another COPD phenotype like small airways disease, chronic bronchitis, or asthma COPD overlap syndrome can also cause hyperinflation and symptoms. Assessment of a low diffusing capacity in LVR candidates requires a lot of nuances, and a Dlco of <20% of the predicted value cannot be regarded as a definite exclusion criterion. Good outcomes have been published in patients with a low (<20% of predicted) Dlco.^24^ Reliably measuring Dlco in patients with severe emphysema is difficult and often does not reflect the real gas exchange properties. It is important to combine the information of the with Pao_2_, chest CT scans, and sometimes perfusion scintigraphy to make a decision on eligibility in patients with low Dlco.^24^

### Step 6: Radiologic Assessment

**Figure 2:**
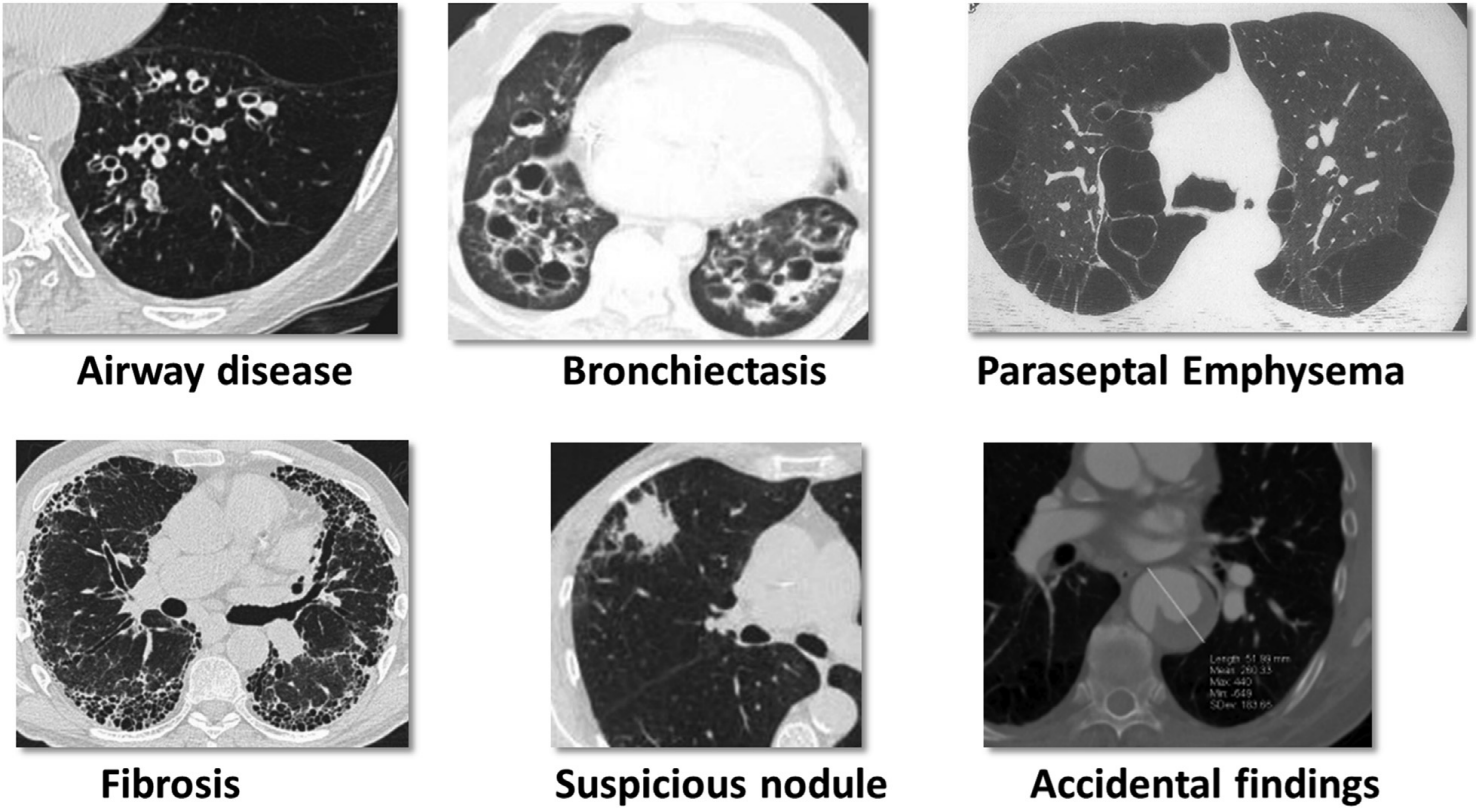
CT findings that exclude patients from endobronchial valve treatment. Each of these findings require a different treatment approach. (Reprinted with permission from Respiration, S. Karger AG, Basel, Switzerland).^4^

If a patient seems to be suitable for EBV treatment according to the step-wise approach, it is recommended first to perform a visual assessment of the HRCT scan to identify possible treatment target lobe(s) and to identify potential concurrent disease that might disqualify a patient for valve treatment (eg, bronchiectasis, paraseptal emphysema, unstable nodule) (Fig 2).

**Figure 3:**
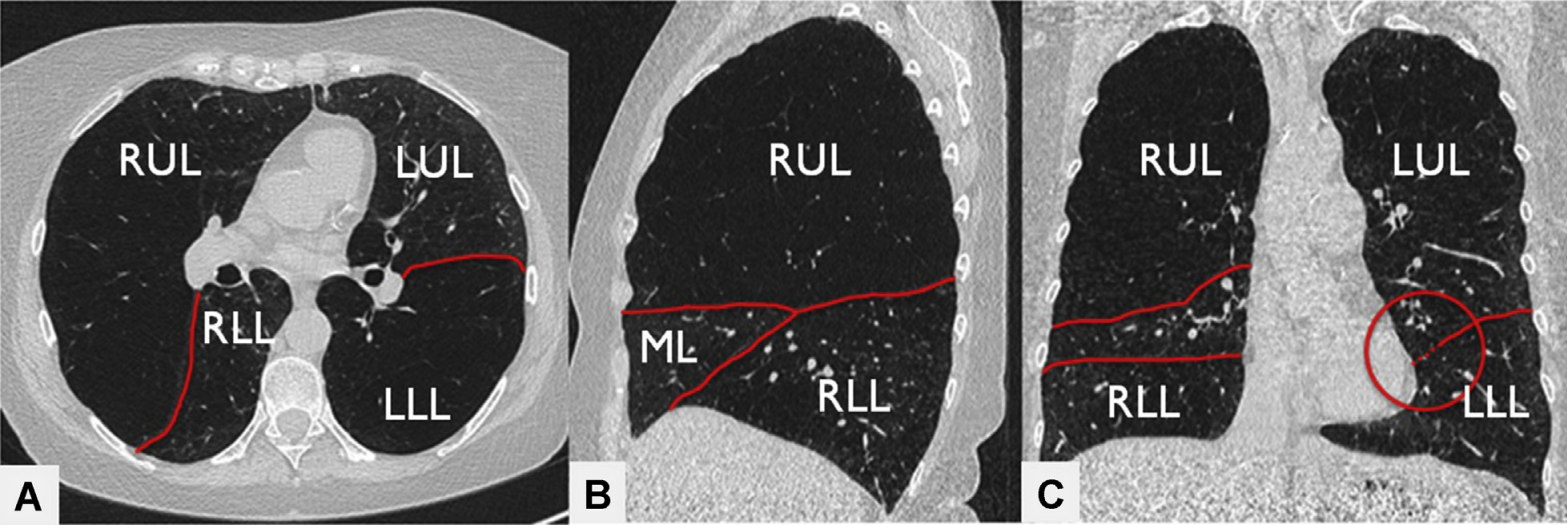
A-C, Example of visual assessment approach of the high-resolution CT scan. A, Axial view; B, sagittal view; C, coronal view of 1 mm high-resolution CT scan reconstructions with the use of a sharp kernel. The red lines indicate the fissures. The right upper lobe shows more destruction compared with the other lobes. The fissures in the right lung look intact on all three views. The left major fissure looks intact in the axial view; however, in the coronal view, a small gap is visible. Based on these slices, with the use of a visual assessment, the right upper lobe is a potential Zephyr endobronchial valve (Zephyr EBV; Pulmonx Corporation, Redwood City, CA) target lobe. LLL = left lower lobe; LUL = left upper lobe; ML = middle lobe; RLL = right lower lobe; RUL = right upper lobe.

Preferably a thin slice (1 mm) noncontrast inspiration and expiration HRCT scan is performed and reconstructed in both a soft kernel (for quantitative CT [QCT] analysis) and a sharp kernel (for visual assessment).^25^ Visual CT assessment can best be performed on all three angles (coronal, sagittal, and axial views). The integrity of the fissures can be assessed roughly, and possible EBV target(s) can be preliminarily identified. An example of visual assessment of the HRCT scan is given in Figure 3.

#### QCT Analysis

**Figure 4:**
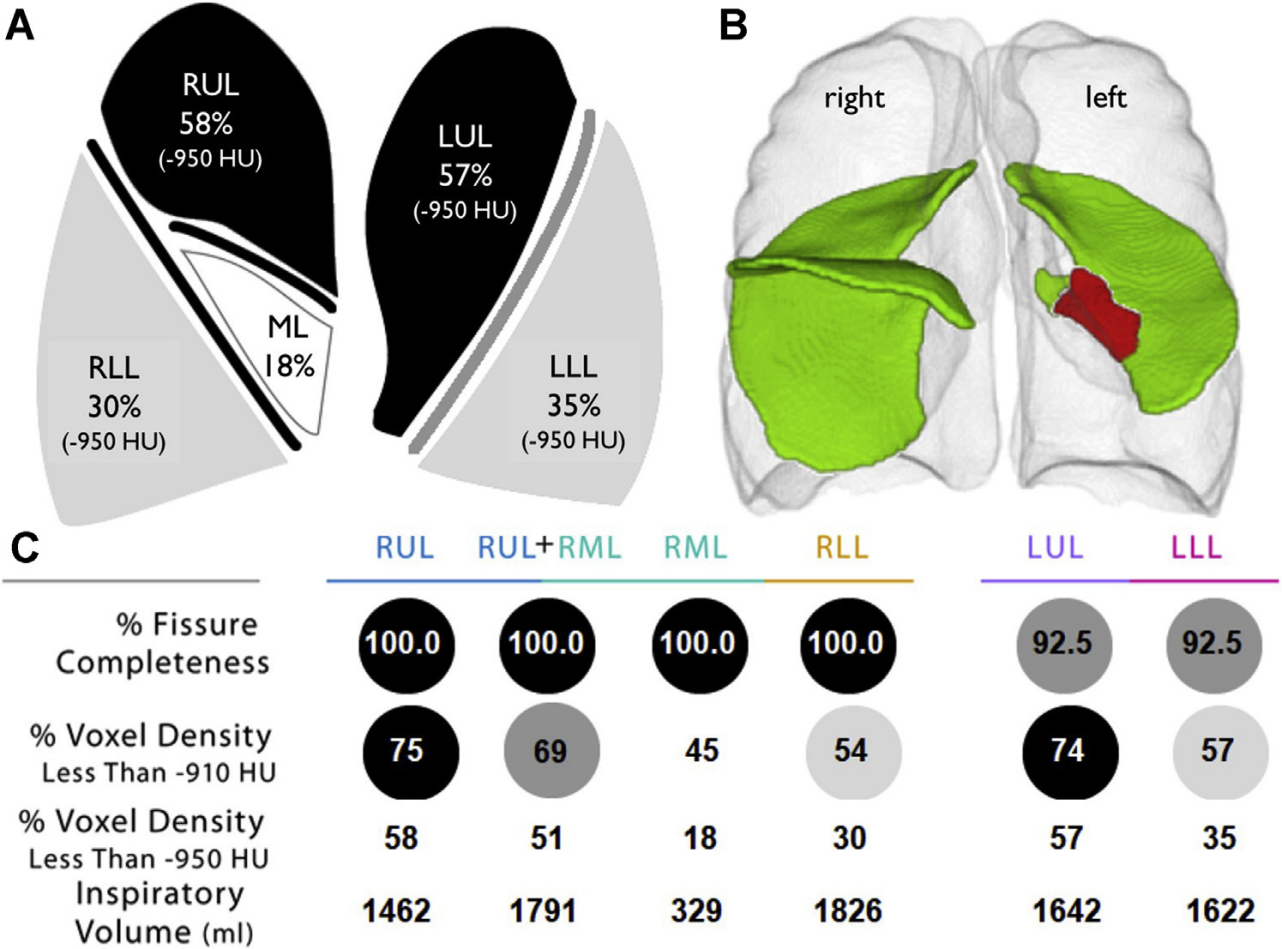
A-C, An example of quantitative analyses of high-resolution CT scan. A. Lung cartoon with per lobe the percentage of low attenuation areas (at −950 Hounsfield Units) values displayed. B. Rendering picture of the fissures. Green color indicates intact fissure; red color indicates a gap in the fissure. In this example, there is a gap visible in the left major fissure. This might imply that there is presence of collateral ventilation between left upper lobe and left lower lobe. C, Results of the percentage of fissure completeness, percentage of low attenuation areas (at −910 and −950 Hounsfield Units), and the inspiratory volumes are summarized per lobe. Based on this quantitative analysis (StratX Lung Report; Pulmonx Corporation, Redwood City, CA), the right upper lobe or the left upper lobe can be confirmed as potential targets for Zephyr endobronchial valve (Zephyr EBV; Pulmonx Corporation, Redwood City, CA). However, collateral ventilation needs to be ruled out by Chartis measurement.^31^ (Published with permission from Pulmonx Corporation). LLL = left lower lobe; LUL = left upper lobe; ML = middle lobe; RLL = right lower lobe; RML = right middle lobe; RUL = right upper lobe.

The next, and presumably most important, step in the assessment of LVR eligibility is to perform a quantitative analyses of the HRCT scan.^26^^,^^27^ QCT analysis is recommended in all patients evaluated for LVR (also shown to be of value for LVR^28^). A QCT analysis provides a summary of the amount of emphysema per lobe, volume per lobe, and a detailed integrity score of all fissures (Fig 4).

A certain amount of emphysematous destruction is necessary to occlude a lobe with valves safely and to obtain clinical benefit. The percentage of low attenuation areas at −950 Hounsfield Units needs to be approximately >30% (or >50% at −910 Hounsfield Units).^29^^,^^30^ If there is not enough emphysematous tissue present in the target lobe, too much functional lung tissue will become atelactatic and cause V/Q mismatch (shunting) with resultant chest discomfort and dyspnea and likely no benefit of the procedure.

Lobar volumes also have to be considered in guiding treatment decisions. As an example, when considering a target lobe with a 2500 mL volume against the ipsilateral lobe with a volume of 750 mL, serious problems could occur if the ipsilateral lobe with a small volume has no healthy lung tissue (eg, destruction scores >30% at −950 Hounsfield Units). No clear cut-off values exist for these volumes, but a judgment would have to be made on whether the smaller remaining lobe is capable of occupying the entire hemithorax again after treatment.

Fissure integrity is a surrogate for the absence or presence of collateral ventilation.^31^, ^32^, ^33^ EBV treatment is effective only if there is absence of collateral ventilation in the target lobe.^1^^,^^5^, ^6^, ^7^^,^^34^ Recent studies have shown that patients with an incomplete fissure based on a fissure completeness score (FCS; with the use of QCT analysis) of <80% can be excluded from Chartis measurement and EBV treatment.^31^^,^^33^

In patients with >80% complete fissures, the FCS is not specific enough for EBV treatment decision. In this case, additional Chartis measurements are always recommended for the right lung fissures.^31^ For the left lung, Chartis assessments may be omitted if the FCS is >95%.^31^ The advantage of always performing Chartis assessments is to ensure that there can be an expected treatment benefit and that any lack of volume reduction after valve placement is most likely not due to the presence of collateral flow but rather due to a valve misplacement or other factors, such as adhesions limiting lung deflation.

#### Treatment Target Lobe Selection

Selecting the best EBV treatment target lobe is crucial for a good outcome and to avoid serious complications such as severe hypoxia, respiratory failure, and severe pneumothorax. Only one lobe can be treated in a single procedure for safety reasons. However, the right upper lobe and right middle lobe combination can be regarded as a single lobe in cases in which there is collateral flow across the minor fissure. Selecting the optimal target lobe requires combining diagnostic information, where both absence of collateral ventilation (Chartis or FCS on CT scan) and at least 30% emphysematous destruction at ≤−950 Hounsfield Units (≥50% at ≤−910 Hounsfield Units) with the use of QCT analysis, are important features to assess. The ideal lobe (and lung) targeted for treatment is characterized by the highest level of emphysema heterogeneity, the lowest perfusion present on nuclear perfusion scintigraphy (or alternative perfusion methods), balanced lung volumes, and most air trapping (with the use of expiratory CT scan, especially helpful in homogeneous cases). Local factors such as significant pleural adhesions or pleural thickening,^35^ presence of bronchiectasis, fibrotic changes, nodules or a large bulla, or significant paraseptal emphysema just adjacent to the target lobe might make a potential target lobe less suitable for treatment. Based on the emphysema distribution on QCT analysis, combined with a treatable FCS, it is possible to have multiple targets for an individual patient. Again, combining visual CT (and air trapping), QCT analysis, perfusion data, and Chartis assessment will result in deciding on the one preferable lobe. In general, for a more homogeneous emphysema distribution, relatively more hyperinflation, and preserved gas exchange (ie, better Dlco and blood gasses) are needed to get a good outcome after treatment, compared with a heterogeneous (upper or lower lobe) emphysema distribution.

## How Do I Treat Patients?

**Figure 5:**
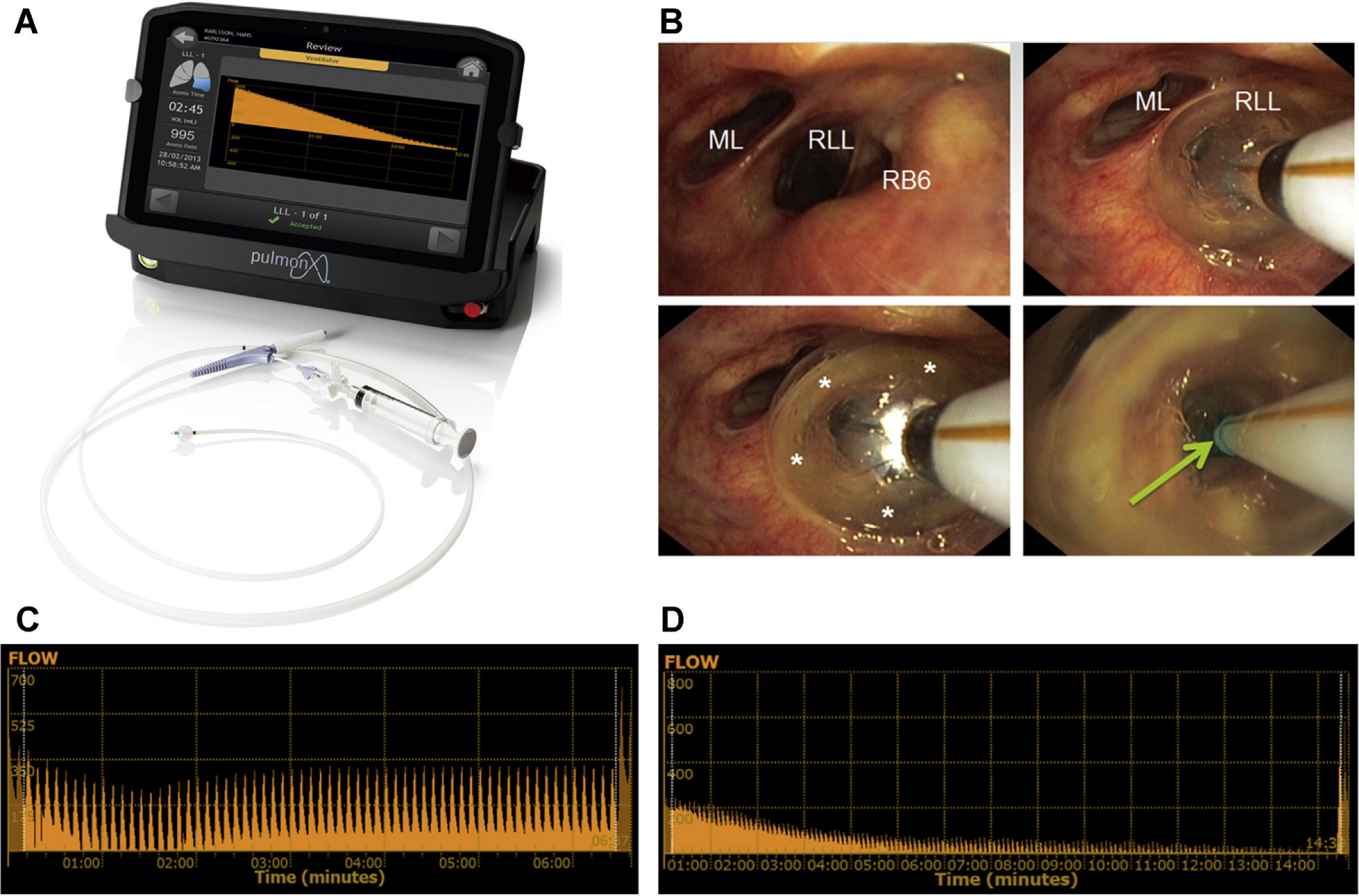
A-D, Chartis Pulmonary Assessment System. A, Chartis Pulmonary Assessment System (Pulmonx Corporation, Redwood City, CA) with Chartis balloon catheter. B, Example of temporary occlusion of the entrance of the right lower lobe with the Chartis balloon catheter. C, A continuous flow reading indicates collateral ventilation in the target lobe; no valves will be placed. D, A gradual decrease of the flow to no flow indicates the absence of collateral ventilation; valves can be placed into the target lobe. ML = middle lobe; RB6 = right lower lobe apical segment; RUL = right upper lobe.

Both the Chartis measurement and the placement of the valves can be performed preferably in a single procedure (to avoid additional bronchoscopy-related adverse events^36^), with deep conscious sedation or with general anesthesia.^3^^,^^37^^,^^38^ In practice, it turns out that both Chartis and the placement of the valves with general anesthesia is more accurate and easier to perform because there is less coughing, less mucus production, and less mucosal swelling and that the diameter of the airways can be measured more precisely to select the correct valve size for placement.^3^^,^^4^^,^^38^ The bronchoscopy itself can be performed according to local practice. A short course of prophylactic antibiotics and prednisolone can be considered around the treatment period.^36^ Both rigid (best with high frequency jet ventilation) and flexible intubation (best with the use of volume controlled positive pressure ventilation, with a low frequency and a long expiration time) are suitable airway access options. Although patients with very severe COPD can be considered as a high-risk group in relation to the appearance of perioperative complications, anesthesia during these procedures does not cause additional adverse events and can be applied safely.^1^^,^^3^^,^^38^ The Chartis System^4^^,^^34^ that comprises the balloon catheter and console provides a physiologic measure of collateral ventilation (Fig 5). With the use of the balloon catheter, the treatment target lobe is fully blocked temporarily, with the lobar air able only to escape through central lumen of the catheter, which is measured by the console. If there is no collateral ventilation to feed the airway distal to the balloon, air flow out of the lobe will gradually decrease. A continuous flow indicates the presence of collateral ventilation in the target lobe. The entire measurement takes approximately 10 to 15 minutes.^3^ The Chartis measurement can also be used as a trial to assess the dependence of the treatment target’s contribution to the entire gas exchange. When using Fio_2_ levels approximately 30% to 40%, blocking the target should not result in a significant desaturation. This can be an important tool, especially in patients with a homogeneous emphysema distribution, low Dlco, or low emphysema destruction scores. If patients experience significant desaturation, selection of the target lobe might have to be reconsidered.^39^

**Figure 6:**
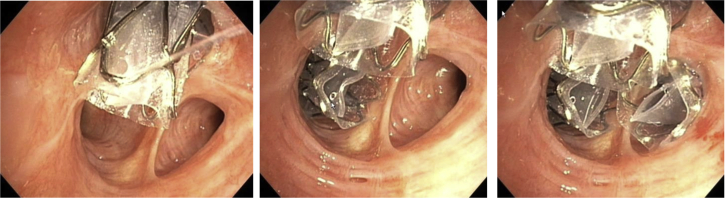
Bronchoscopic view example of a right upper lobe treatment with endobronchial valves shows a full lobar occlusion to achieve the desired atelectasis of the lobe with consecutive valves placed in RB1, RB3, and RB2 (left to right).

**Figure 7:**
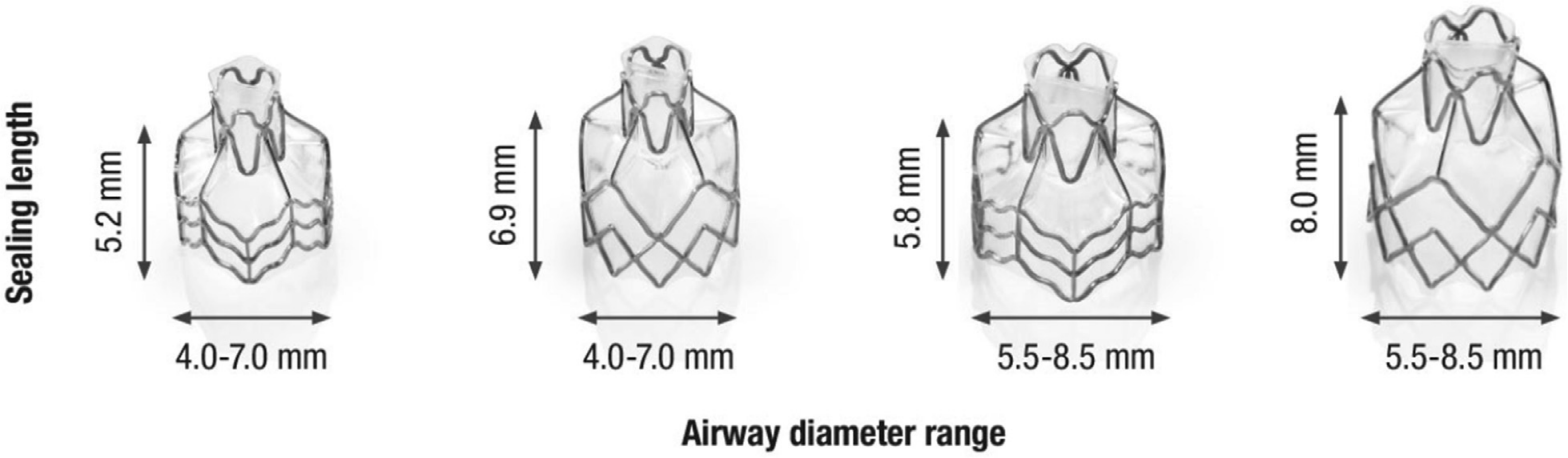
The four available endobronchial valve sizes (Zephyr EBV; Pulmonx Corporation, Redwood City, CA).

Once the absence of collateral ventilation has been established, the valves are placed in all (sub-) segments of the target lobe. Depending on airway anatomy, normally three to five valves are placed (Fig 6). The EBVs (Zephyr-EBV, Pulmonx Corporation, Redwood City, CA) adapt to the dynamic conditions of the airway; for airway sizing (with the use of the markers on the delivery catheter) just before placement, it is necessary to choose one of the four valve sizes that are available (Fig 7).

**Figure 8:**
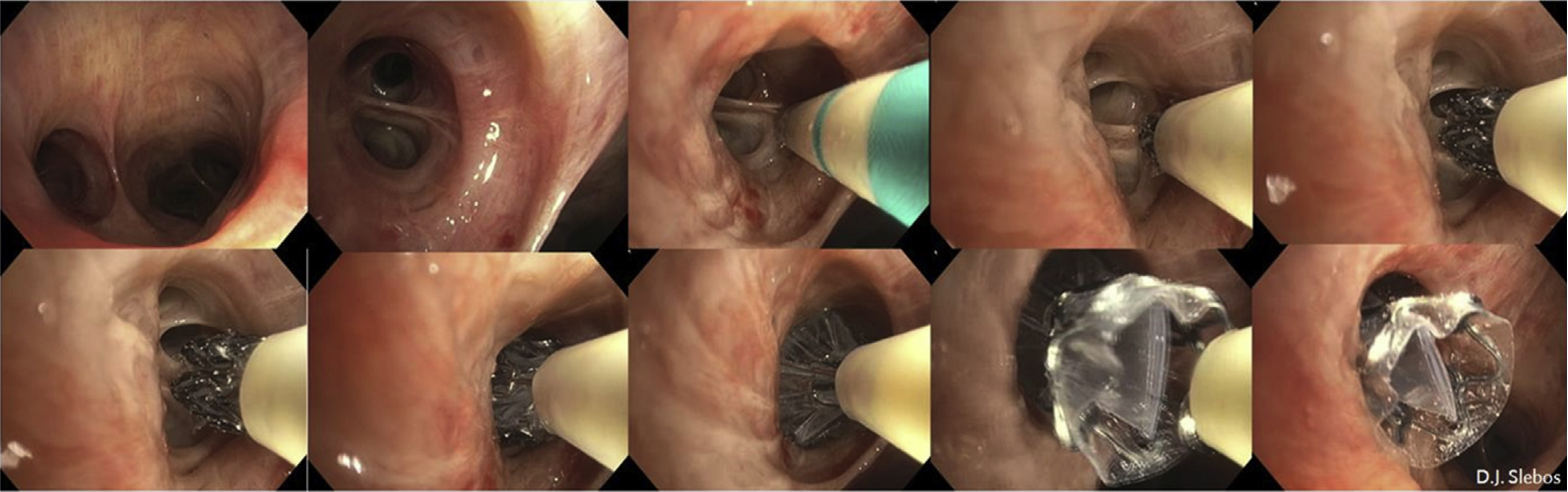
Endobronchial valve placement example of the LB6 (apical segment of the left lower lobe). Clockwise, from left to right: left lower lobe entrance image, view of the LB6 with the Zephyr endobronchial valve (Zephyr EBV; Pulmonx Corporation, Redwood City, CA) catheter placed against the next subcarina indicates the length for a short size valve and subsequent deployment of the valve against this subcarina, which on further release expands against the LB6 wall, sealing it completely. The last picture shows the Zephyr endobronchial valve in place, with just the Heimlich valve part visible.

The required valve is loaded into the delivery catheter that will be inserted into the working channel of the bronchoscope. The valves are placed into the desired segmental airway by deploying the valve, under direct vision, that is positioned at the middle of the next subdivision carina, or if not visible enough, with the use of the valve-length markers on the dedicated EBV catheter to align the valve with the proximal carina (Fig 8). It is important not to treat the entire lower lobe bronchus (B7-10) with just one valve, because airway dynamics at this location are just too great and may cause valve leakage or dislocation.^4^ Our local practice to prevent severe coughing immediately after the procedure involves a single application of local lidocaine (1% vol/vol) at the treatment lobe and a systemic dosage of opioids (eg, morphine, fentanyl) before extubation and lidocaine (30 to 50 mg 1% vol/vol); this can be repeated the first hours if needed. No further anticough agents are given.

Once all valves have been placed, the procedure is complete, and the patient must be observed closely for pneumothorax occurrence (which, based on the previous studies, is approximately 20%).^40^^,^^41^ In our hospital, patients are observed for 2 hours on the recovery ward, where bedside chest radiography is performed. If there are no adverse events, the patient is transferred to the pulmonary ward for at least three nights after the treatment for observation. It is important to make sure that the ward staff are fully aware and that it is clearly documented in the medical charts that the admitted patient is a valve patient at potential risk for a pneumothorax. To facilitate awareness, we also provide our patients with a warning bracelet. Emergency chest tube facilities should be available, and staff must be able to insert a chest tube in this situation 24/7 (in our hospital, we run regular training sessions on emergency chest tube placement, just to make it a standard routine). In our practice (not supported by scientific evidence) on the treatment day, the patient has “bed-rest,” and only a walk to the toilet is allowed. The days thereafter, the patient is mobilized. On the first day and three days after the treatment, chest radiography is performed, with additional radiograms, depending on symptoms. If the patient’s condition is stable without complaints, and no pneumothorax is visible on the chest radiogram, the patient is discharged. Patients receive clear verbal instructions and written information on symptoms of a pneumothorax and what to do if complaints occur.

## How Do I Follow Up With Patients?

Dedicated follow up of patients is important in optimizing treatment outcomes. In our hospital, we keep track of our treated patients by using the following scheme: One week after discharge, a telephone consultation is performed; at 6 weeks, the patient is invited to the hospital for medical assessment, chest CT scan, and pulmonary function tests. If a patient does not experience clinical benefit and the CT scan shows no lobar atelectasis of the treated lobe, a revision bronchoscopy is scheduled to “fine-tune” the treatment by replacing any mispositioned valves.^40^ After this, patients will be invited for an in-office assessment at 6 months, 1 year, and annually up to the 5-year follow up. We also record all the patient data captured in our national BLVR registry (BREATHE-NL Registry, NCT02815683).^42^

## How Do I Manage Adverse Events?

A LVR treatment can be associated with adverse events. Valve treatment is virtually a “bronchoscopic lobectomy,” and the treating physician should be able to deal with the complications and challenges involved.^40^ Complications can include, most importantly, the occurrence of pneumothorax (15% to 25% of the treated patients),^41^ and in rare cases postobstruction pneumonia, severe hemoptysis, airway kinking, hypoxia due to shunting, and persistent cough (all ±<2%).^40^^,^^43^ Furthermore the treatment can result in lack of initial benefit due to misplaced valves, an untreated (sub-) segment, or presence of collateral ventilation (collateral ventilation not assessed prior to valve placement or false positive result). Next to this, the achieved initial benefit may dissipate over time due to granulation tissue formation.^40^^,^^44^

The majority of pneumothoraces occur within 72 hours after treatment.^4^^,^^5^^,^^41^^,^^45^^,^^46^ In most symptomatic cases, simple intercostal drainage is sufficient to manage the pneumothorax. In situations in which there is a persistent high-flow air leak from a proper functioning intercostal drain, temporary removal of a single valve should be considered (which can be replaced after 6-8 weeks). If this still does not resolve the issue, removal of all valves might be considered.^41^ Normally, the outcome after a pneumothorax after valve treatment is good.^45^

Further guidance on the management of complications after BLVR with the use of one-way valves can be found in the recently published expert review.^40^

## How Do I Organize My BLVR Treatment Program?

Basic elements for a successful program are dedication, COPD expert knowledge, access to interventional pulmonology, access to multimodality treatments for COPD (other bronchoscopic and surgical LVR techniques,^47^ pulmonary rehabilitation, noninvasive ventilation, and lung transplantation),^21^^,^^48^ well-organized in-house logistics and facilities (radiology, pulmonary function testing, anesthesia, thoracic surgery), and a solid referral network. Participation in scientific efforts can be important for advancing the BLVR field and getting the exposure needed for patient referrals. Also, it is highly recommended that all patient treatments and outcomes are captured in a (national-) registry, especially for quality control reasons.^42^ Furthermore, it is strongly advised to discuss treatment strategies for patients with severe COPD in a multidisciplinary team, similar to organized the care for patients with interstitial lung disease and lung cancer. Only by doing this can you ensure the best possible treatment is offered to each individual patient with COPD.^21^^,^^42^^,^^48^ As an expert center, we offer other hospitals the chance to discuss potential cases using a weekly scheduled videoconferencing meeting. By organizing this, we educate referral and other treatment hospitals to prevent them from the pitfalls we previously encountered, thereby ensuring high quality standards. Additional training, attending our program, or proctoring of cases can be the result of this interaction. Sharing knowledge among novices and experts worldwide is key to improving this field of medicine further.

The systematic approach to careful patient selection, proper procedural technique, before and after treatment, and dedicated long-term follow up allows for good clinical outcomes and respectful application of this important interventional treatment of patients with severe COPD with hyperinflation.
